# Microfluidic innovations in chronic kidney disease and renal fibrosis: from mechanistic insights to clinical applications

**DOI:** 10.3389/fmed.2026.1723501

**Published:** 2026-02-10

**Authors:** Anqi Liu, Kun Xiao, Hongli Lin

**Affiliations:** 1 General Ward, Guizhou Provincial People’s Hospital, Guiyang, Guizhou, China; 2 Department of Nephrology, The First Affiliated Hospital of Dalian Medical University, Dalian, Liaoning, China

**Keywords:** biomarker detection, chronic kidney disease, drug screening, kidney-on-a-chip, microfluidics, renal fibrosis

## Abstract

**Background:**

Chronic kidney disease (CKD) and renal fibrosis remain major global health burdens, with limited options for early diagnosis and effective therapy. Conventional approaches, such as kidney biopsy and imaging, are invasive or insensitive to early-stage changes. Microfluidic technology has emerged as a transformative platform that enables precise modeling of renal microenvironments, sensitive biomarker detection, and physiologically relevant drug testing. This review evaluates recent advances in microfluidics for CKD and fibrosis, with emphasis on mechanistic insights, diagnostic innovations, and therapeutic strategies.

**Discussion:**

Mechanistic studies using organ-on-a-chip systems, including glomerulus- and tubule-on-a-chip, have replicated critical pathophysiological processes such as proteinuria-induced podocyte injury, epithelial–mesenchymal transition, FAO dysregulation in tubular cells, and immune cell-mediated inflammation. These models provide superior resolution compared with 2D culture or animal models and have identified novel fibrotic pathways—how they work: by perfusing media through microchannels to simulate shear stress; advantages: dynamic real-time monitoring; disadvantages: high cost and limited throughput; limitations: often lack full multi-cellular integration; translational value: patient-specific modeling for precision nephrology. Diagnostic innovations include microfluidic biosensors for non-invasive, high-sensitivity detection of CKD biomarkers such as albumin and neutrophil gelatinase-associated lipocalin (NGAL), as well as multiplex platforms that analyze multiple analytes in urine or blood simultaneously. Wearable epidermal patches have further extended applications to continuous monitoring of electrolytes and metabolites, enhancing patient-centered management. Therapeutically, microfluidic systems support high-throughput drug screening under physiologically relevant perfusion, enabling more predictive antifibrotic testing. Microfluidic-assisted nanodelivery platforms improve drug targeting and bioavailability, while organoid-on-chip systems enhance stem cell differentiation and regenerative potential. Integration with artificial intelligence and multi-omics further refines data interpretation, biomarker discovery, and personalized therapy design.

**Conclusion:**

Microfluidic technologies bridge the gap between bench and bedside by enabling mechanistic discovery, sensitive biomarker detection, and translational therapeutic testing in CKD and fibrosis. Despite significant advances, challenges remain in scalability, reproducibility, and regulatory approval. Addressing these hurdles through interdisciplinary collaboration will be essential. With continued innovation, microfluidic systems hold strong promise for advancing precision nephrology and improving patient outcomes.

## 1 Introduction

The Global Burden of Disease Study 2023 estimates that chronic kidney disease (CKD) affects 788 million adults worldwide in 2023, causing 1.48 million deaths and 52.4 million disability-adjusted life years, primarily driven by metabolic risks like diabetes and hypertension, with the fastest growth in low- and middle-income regions (1–4). Renal fibrosis is central to the progression of CKD and is characterized by excessive extracellular matrix (ECM) deposition, tubular atrophy, and microvascular rarefaction, which together disrupt renal architecture and function (5, 6). Despite advances in understanding molecular drivers such as transforming growth factor-beta (TGF-β) signaling, epithelial-mesenchymal transition (EMT), dysregulated fatty acid oxidation (FAO) in tubular cells, and immune cell crosstalk, clinical translation is hindered by the lack of early diagnostic tools and therapies capable of halting or reversing fibrotic processes (7). Traditional diagnostic methods, including invasive kidney biopsies and indirect imaging modalities like shear wave elastography (SWE) or diffusion-weighted MRI (DWI), suffer from sampling bias, low sensitivity in early stages, and an inability to differentiate active inflammation from irreversible scarring (8, 9). Similarly, current antifibrotic strategies—ranging from renin-angiotensin system inhibitors to experimental agents targeting TGF-β—often fail to address the spatial and temporal complexity of fibrotic microenvironments (10, 11).

Microfluidic technology has emerged as a transformative platform, potentially overcoming these limitations. By enabling precise replication of renal tissue interfaces, dynamic fluidic control, and real-time monitoring at single-cell resolution, microfluidic systems bridge the gap between traditional 2D cell cultures and *in vivo* models (12, 13)—how this works: controlled flow in chips mimics physiological conditions; advantages: superior resolution and ethical reduction of animal use; disadvantages: fabrication complexity; limitations: scalability issues; translational value: accelerates biomarker validation like neutrophil gelatinase-associated lipocalin (NGAL) for early CKD detection. For instance, organ-on-a-chip (OoC) platforms, such as the Fluidic 480 system (commercially available from vendors like Emulate Bio), allow the co-culture of tubular epithelial cells, fibroblasts, and endothelial cells under physiologically relevant shear stress, thereby recapitulating the interplay between hypoxia, metabolic dysfunction (e.g., FAO crosstalk), immune cell crosstalk (e.g., macrophage-mediated signaling), and fibrotic pathways (14, 15). These models also facilitate high-throughput drug screening, as demonstrated in studies testing novel inhibitors of pro-fibrotic pathways (16, 17). Furthermore, microfluidics-based biosensors have shown promise in detecting CKD biomarkers like NGAL at femtomolar sensitivity, offering non-invasive alternatives for early diagnosis and disease monitoring (18–21).

**FIGURE 1:**
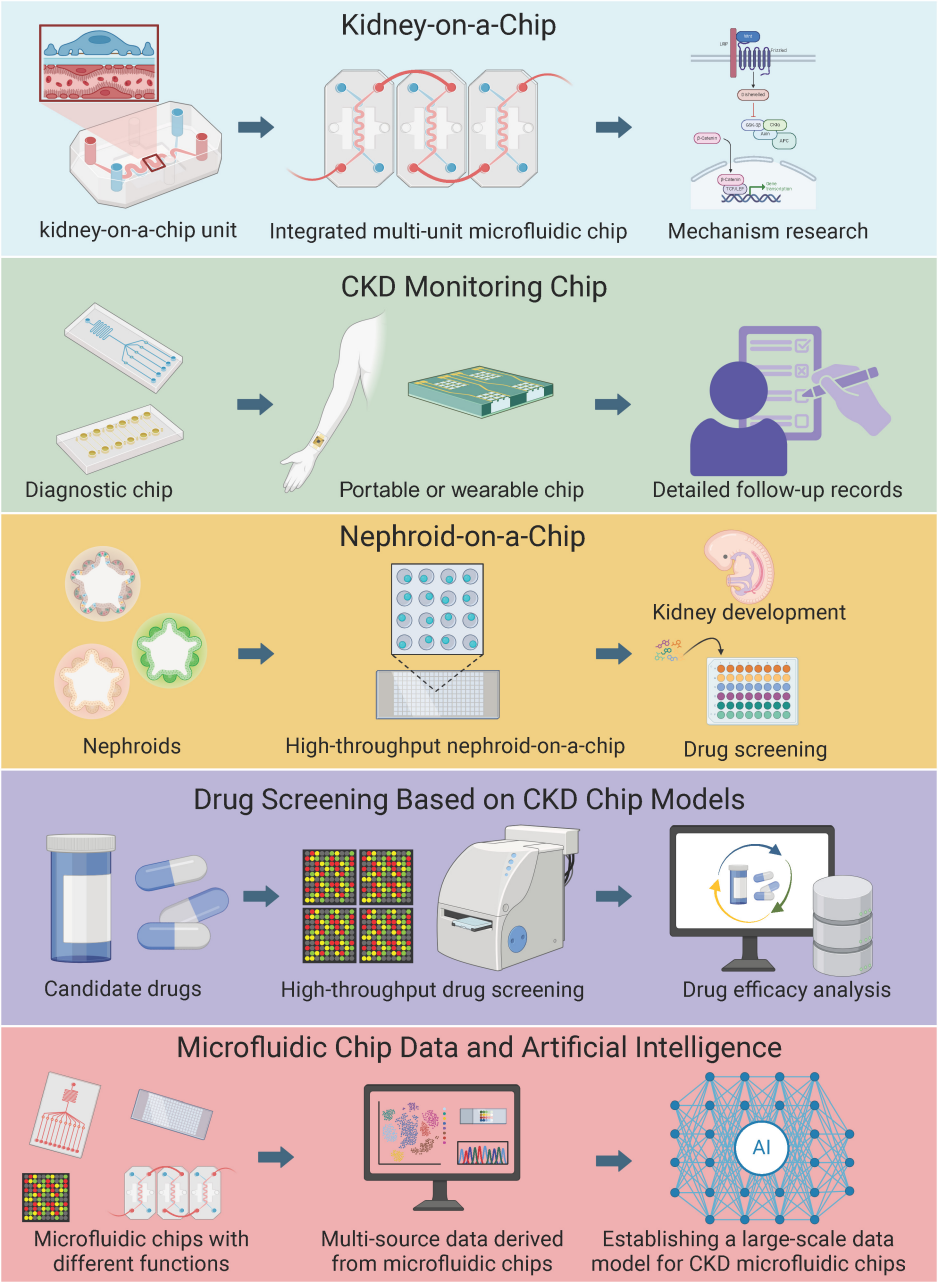
Applications of Microfluidic platforms in chronic kidney disease and renal fibrosis. This schematic illustrates a multi-layered framework of microfluidic innovations, from basic modeling to advanced clinical and data-driven applications. The top layer depicts the foundational kidney-on-a-chip (KoC) unit, progressing to an integrated multi-unit microfluidic chip dedicated to mechanistic research in renal pathophysiology. The second layer focuses on CKD monitoring chips, evolving from diagnostic chips to portable or wearable devices for detailed, real-time patient follow-up records. The third layer represents nephroid-on-a-chip platforms, starting with nephroids and advancing to high-throughput nephroid-on-a-chip systems for studying kidney development and initial drug screening. The fourth layer highlights drug screening based on CKD chip models, involving candidate drugs, high-throughput screening processes, and subsequent drug efficacy analysis. The bottom layer integrates multi-source data from microfluidic chips with diverse functions, culminating in the establishment of a large-scale data model for CKD using artificial intelligence to support research and therapeutic strategies. Created with Canva.com.

This review synthesizes recent advances in microfluidic applications for CKD and renal fibrosis research, focusing on three main areas: (1) mechanistic insights into fibrosis drivers, including metabolic reprogramming and immune cell crosstalk; (2) diagnostic innovations through biomarker detection and organ-on-chip models; and (3) therapeutic breakthroughs in drug delivery and regenerative medicine. By integrating interdisciplinary approaches, such as AI-driven analysis of microfluidic datasets and CRISPR-edited organoids, we highlight how microfluidics is redefining precision nephrology and accelerating the translation of bench discoveries to clinical interventions (Figure 1)—critically, while promising, gaps in multi-organ modeling persist. For clarity, studies cited as 2024–2025 publications refer to articles available as early online releases or accepted manuscripts at the time of writing; no unpublished or non–peer-reviewed data are included.

## 2 Pathological mechanisms of renal fibrosis: Insights enabled by microfluidics

While this review primarily focuses on renal fibrosis, selected examples from non-renal systems are included solely to illustrate transferable microfluidic design principles or analytical strategies, rather than to suggest direct mechanistic equivalence across organ systems. Renal fibrosis, a hallmark of CKD progression, involves complex molecular, cellular, and biomechanical interactions leading to ECM deposition and irreversible tissue scarring. Traditional models often fail to capture the spatiotemporal complexity of fibrotic microenvironments. However, microfluidics has emerged as a transformative tool for dissecting these mechanisms with exceptional resolution (Figure 1). A pivotal example is the glomerulus-on-a-chip model developed by Musah et al. This system utilized human induced pluripotent stem cell (iPSC)-derived podocytes and human glomerular endothelial cells cultured on opposite sides of a permeable membrane under physiological fluid shear stress (0.5 dyn/cm^2^). The technology works by perfusing media through microchannels to mimic glomerular filtration, allowing real-time monitoring of barrier function via albumin permeability assays. Experiments exposed the model to hyperglycemic conditions (30 mM glucose, 7 days), revealing podocyte injury (40% nephrin loss) and barrier breakdown—contributing to the field by providing the first human-relevant model of diabetic nephropathy mechanics, superior to animal studies for species-specific insights (22, 23).

To model proteinuria-induced tubulointerstitial fibrosis, Liu et al. established a sophisticated microfluidic co-culture system integrating human proximal tubular epithelial cells (PTECs) with human glomerular endothelial cells (GECs) in adjacent channels. The platform operates via controlled shear stress (1.5 dyn/cm^2^) and albumin overload (40 mg/mL, 48 h) through parallel microchannels, with qPCR and immunofluorescence quantifying EMT (2.5-fold α-SMA upregulation) and endothelial inflammation. This contributed by elucidating paracrine crosstalk, a mechanism elusive in 2D cultures, advancing fibrosis pathway understanding (24).

Microfluidics also excels in modeling vascular aspects. For instance, Kim et al. utilized a glomerulus-on-a-chip platform perfused with serum from patients with glomerulonephritis, identifying circulating factors compromising barrier integrity via live-cell imaging (50% permeability increase) (25). Furthermore, microfluidic chips have been utilized to create kidney-on-a-chip platforms that simulate hydronephrosis and facilitate molecular pathological analysis (26). In the context of kidney disease research, microfluidic chips have been used to assess functional changes in renal tubular cells under varying microenvironments, revealing enhanced expression of ion transporters and drug-metabolizing enzymes via transcriptomic and proteomic analyses (27). However, this study’s reliance on animal-derived Madin-Darby canine kidney (MDCK) cells limits direct translational relevance to human CKD; future work should prioritize human iPSC or primary cells for clinical applicability. Additionally, microfluidic technology simulates the passage and obstruction of malaria-infected red blood cells, providing quantitative insights into the infection process (28).

To address metabolic crosstalk in CKD, recent studies have highlighted FAO dysregulation in tubular cells under hypoxia, showing mitochondrial metabolic reprogramming (reduced CPT1A activity leading to lipid accumulation and fibrosis), emphasizing crosstalk between metabolic pathways and fibrotic signaling (29, 30). For example, while metabolomics has been used in kidney research for spatial profiling [e.g., via MALDI trapped ion mobility imaging mass spectrometry to localize metabolites like argininic acid and acetylcarnitine to renal structures (31)], integration with microfluidics remains limited, primarily focusing on general lipid dysmetabolism rather than specific FAO simulation in tubule-on-a-chip setups; advantages of potential integration include real-time metabolite tracking absent in static models, but limitations involve oversimplification of systemic influences and limited commercial adaptations; translational value lies in identifying metabolic therapy targets, though full microfluidics-metabolomics integration in kidney models is a key knowledge gap. For immune cell crosstalk, Gijzen et al. established an immunocompetent KoC co-culturing PTECs with primary monocytes, demonstrating how complement-activated human serum induces kidney inflammation through monocyte extravasation and epithelial damage (32). Drawing from successes in liver fibrosis models [e.g., Lee et al., bioprinted hepatocytes activating stellate cells via paracrine TGF-β (33)], kidney adaptations could incorporate similar spatial controls to better model fibroblast activation in renal fibrosis—providing contextual comparison without overshadowing CKD focus. Critically, these studies summarize microfluidics’ shift from basic modeling to advanced, multi-cellular systems, yet knowledge gaps in long-term fibrosis progression persist, urging hybrid *in vivo*-microfluidic validations.

## 3 Diagnostic innovations via microfluidics

The limitations of conventional diagnostic approaches for CKD and renal fibrosis—including invasiveness, low sensitivity in early stages, and inability to capture dynamic disease progression—have driven the development of microfluidic technologies. These platforms provide significant advantages in biomarker discovery, functional assessment, and point-of-care testing (POCT), transforming how clinicians detect, monitor, and stratify fibrotic kidney diseases (Table 1).

**TABLE 1:** Representative microfluidic biosensors for chronic kidney disease (CKD) diagnostics.

Biomarker(s) detected	Sample type	Microfluidic platform/ chip/model	Cell types/origin	Key finding	Limitations	References
Albumin (urinary)	Urine	Passive mixing urinary albumin chip	N/A	Improved reproducibility over dipsticks	Validation in large cohorts needed	(21)
Multi-protein panel (incl. NGAL, MMP-7)	Urine	Microfluidic-MS integration	N/A	High-throughput early fibrosis patterns	High cost and complexity	(35)
Electrolytes and DKK3	Sweat	Wearable epidermal patch	N/A	Continuous real-time fibrosis monitoring	Sweat variability in patients	(36, 37)
miR-21	Urine/Blood	Paper-based DNA biosensor	N/A	Femtomolar early marker detection	Clinical cohort validation required	(18)
Nephrolithiasis markers and Galectin-3	Urine	Microfluidic chip-capillary electrophoresis	N/A	Rapid on-site fibrosis links	Resource-limited applicability	(38)

### 3.1 Non-invasive biomarker detection

Microfluidic biosensors enhance sensitivity for CKD markers, addressing the limitations of traditional methods by enabling rapid, low-volume analysis that captures early fibrotic changes. Key biomarkers relevant to CKD and renal fibrosis include albumin (indicating glomerular damage), neutrophil gelatinase-associated lipocalin (NGAL; a marker of tubular injury with high sensitivity for early AKI progression in CKD, correlating with eGFR decline), and emerging fibrosis-specific ones like matrix metalloproteinase-7 (MMP-7; elevated in ECM remodeling during fibrosis), monocyte chemoattractant protein-1 (MCP-1; promoting inflammation and fibroblast activation), and Dickkopf-3 (DKK3; predicting fibrosis progression via Wnt signaling inhibition, with urinary levels directly correlating to renal decline as shown in 2025 studies) (31, 34).

Wu et al. passive-mixing chip detected urinary albumin with better reproducibility than dipsticks (RSD < 5%), demonstrating how microfluidics improves accuracy for early detection (21). Integration with mass spectrometry profiles proteomes, identifying multi-biomarker panels (35). Recent 2025 advancements, such as those in PMC studies, incorporate MMP-7 and DKK3 detection in multiplex platforms, achieving femtomolar sensitivity and enabling comprehensive fibrosis assessment. Wearables aid CKD management, e.g., low-salt monitoring (36). Recently, wearable epidermal patches incorporating microfluidic channels and microvalves allowed continuous, non-invasive monitoring of electrolytes and metabolites, offering real-time assessment of salt intake and fluid balance in CKD patients (37). Guo etal. developed a microfluidic chip-capillary electrophoresis (CE) device with integrated contactless conductivity detection and dynamic multi-segment standard addition for rapid, on-site identification of nephrolithiasis markers (citrate, oxalate, calcium, uric acid) in urine. The system achieves high-throughput analysis in under 3 min with low detection limits (in the μM range), improved repeatability of migration times (RSD < 5%), and effective elimination of matrix effects through on-chip calibration, offering a portable alternative to traditional methods for bedside screening of kidney stone risk (38). Critically, these innovations summarize a shift to multiplex, wearable diagnostics, but standardization gaps hinder widespread adoption.

### 3.2 Organ-on-a-chip for functional assessment

While iPSCs and organoids are emphasized in KoC models for their patient-specific personalization and 3D structural complexity, other sources like primary human renal cells provide mature, functional phenotypes but suffer from limited availability and scalability issues; cell lines offer high reproducibility and ease of use yet lack physiological accuracy. For example, Sakolish et al. utilized primary proximal tubular epithelial cells (PTECs) in a reusable KoC to model nephrotoxicity, revealing more sensitive responses than cell lines (39).

KoC models integrate microfluidic technology with human cells to simulate dynamic renal physiology, providing a critical advantage over static 2D cultures or animal models by incorporating shear stress and real-time monitoring—how this is important: it replicates *in vivo* fluid dynamics, revealing functional responses like transporter activity and barrier integrity that are masked in non-perfused systems, thus offering more predictive data for CKD progression and drug effects; why it matters clinically: it reduces animal experimentation (aligning with 3R principles) and enables personalized assessments using patient-derived cells, accelerating translation from bench to bedside (40). These human-focused microfluidic models align with recent developments in phasing out animal experiments, embodying the 3R principles (Replacement, Reduction, Refinement) as outlined in NC3Rs 2024 guidelines and AMA 2024 revisions—critical for clinical application, as they provide predictive preclinical data that minimizes ethical concerns and improves translation in nephrology (41).

A specific experiment by Homan et al. applied physiological shear stress (0.3 dyn/cm^2^ for 14 days) to iPSC-derived kidney organoids in co-culture with vasculature (human glomerular endothelial cells) in a KoC platform, using RNA-seq and functional assays (e.g., albumin uptake) to demonstrate enhanced maturation: key findings included 3-fold upregulation of tubular transporters (e.g., SLC22A6/OAT1) and improved nephron architecture compared to static controls, highlighting how flow-induced mechanotransduction promotes physiological relevance (42). This is superior to traditional models because it captures real-time metabolic shifts (e.g., FAO responses in CKD-like hypoxia), which animal studies often overlook due to species differences.

Another example is Jang et al. nephrotoxicity experiment in a PTEC-based KoC: cells were exposed to cisplatin (10 μM for 72 h) under perfusion, with live-cell imaging and biomarker assays revealing earlier glutathione depletion and apoptosis (at 24 h) than in static cultures (48 h), underscoring why microfluidics is essential—it mimics shear-induced drug transport, predicting human responses more accurately than rodent models (43). Recent 2024–2025 advancements, such as Emulate Bio’s KoC for functional drug screening, co-cultured liver and kidney compartments to assess repeated-dose toxicity: experiments profiled cytokine release (e.g., IL-6 surge) and barrier function (TEER drop by 40%), identifying nephrotoxic compounds with 85% concordance to clinical data (44, 45). Lin et al. introduced a microfluidic chip-based co-culture system combining human liver spheroids and kidney proximal tubule equivalents for repeated-dose multi-drug toxicity testing, demonstrating enhanced prediction of inter-organ drug metabolism and nephrotoxicity (e.g., aspirin and nicotine interactions leading to biomarker changes like NGAL elevation), with advantages in physiological relevance and reproducibility over static cultures, though limited by throughput; translational value lies in reducing animal testing and supporting preclinical CKD drug evaluation (46).

In terms of biomarkers, KoC facilitates functional validation: for instance, recent studies highlight DKK3 as a predictor of fibrosis progression through Wnt/β-catenin-dependent mitochondrial dysfunction, showing its role in ECM deposition via MFF-mediated mechanisms (31); why important: it bridges mechanistic insights (e.g., Wnt pathway inhibition) to clinical monitoring, outperforming traditional biomarkers like eGFR for early detection; including NGAL for tubular injury (34). Overall, these experiments exemplify microfluidics’ transformative potential, though standardization across labs remains a challenge—critically, summarizing 2024–2025 trends, KoC is evolving toward immunocompetent, multi-organ systems for holistic nephrology advances.

Notably, industry leaders are actively resolving these hurdles: Emulate Bio’s commercially available Kidney-Chip platform, updated with the 2024 Chip-R1 Rigid Chip (constructed from low-drug-absorbing plastics for enhanced ADME and toxicology modeling), integrates real-time sensors for TEER and oxygen, improving throughput and compatibility with advanced data tools (47, 48). These developments make OoC more accessible and scalable for clinical translation. Importantly, although microfluidic and organ-on-a-chip platforms provide improved physiological relevance compared with conventional *in vitro* systems, most current applications should be regarded as hypothesis-generating rather than clinically predictive. Their primary value lies in mechanistic interrogation and preclinical prioritization, and not in direct clinical decision-making without further validation in longitudinal human studies.

### 3.3 Challenges and clinical translation

Microfluidic platforms hold immense potential in CKD diagnosis and treatment, yet clinical translation grapples with multifaceted challenges. Regulatory pathways for OoC systems include FDA’s ISTAND Pilot (2024), qualifying technologies like liver-on-a-chip for DILI prediction, and the Modernization Act 2.0 (2025), easing animal testing mandates to favor human-relevant models (41). Barriers to adopting microfluidic diagnostics encompass quality control (e.g., GMP compliance for sterility), standardization (ISO guidelines for reproducibility), and reimbursement (high costs deterring insurers) (49, 50). Manufacturing hurdles for scalable chips involve material compatibility (e.g., low gas permeability in polymers limiting cell viability), high failure rates in complex fabrication, and elevated costs from low-volume production—addressed partially by 3D printing but still challenging (51, 52). Examples: technologies in clinical testing include the uCR-Chip (2025) for point-of-care creatinine, showing accuracy in trials for CKD staging; versus proof-of-concept like Nature’s 2024 wearable sensors for continuous monitoring, validated in labs but awaiting cohorts (44, 53, 54). Bridging these requires public-private partnerships and clear FDA roadmaps. Critically, these challenges highlight knowledge gaps in cost-effective scaling, balanced by advantages in personalized care.

## 4 Therapeutic strategies enhanced by microfluidic platforms

Microfluidic high-throughput drug screening is a rapidly advancing field that leverages the unique capabilities of microfluidic technology to enhance the efficiency and effectiveness of the drug discovery process. Microfluidic devices enable precise manipulation of minute liquid volumes, significantly reducing the cost and time associated with traditional drug screening methods (Figure 1). These devices are particularly well-suited for high-throughput screening (HTS) as they can integrate multiple assay methods in a compact and automated format. A key advantage of microfluidic platforms is their ability to more closely mimic physiological conditions compared to traditional two-dimensional culture systems. This capability is crucial for developing predictive preclinical models, thereby improving the reliability of drug screening outcomes. For example, microfluidic devices have been used to create organ-on-a-chip models that replicate the complex microenvironments of human tissues, providing a more accurate representation of drug interactions with biological systems (55, 56). A prime example of the power of integrated microfluidic HTS is the work by Azizgolshani et al. (17). They developed a programmable platform accommodating multiple organ-on-chip models with integrated real-time sensors for transepithelial electrical resistance (TEER) and oxygen sensing. This system enabled the unattended, parallel screening of a compound library against a kidney tubule model, with continuous functional monitoring. It successfully identified compounds that ameliorated drug-induced barrier dysfunction, while also flagging those that caused cytotoxic or hypoxic stress. This platform addresses a major bottleneck by automating and integrating functional assays, though its throughput, while high for OoCs, is still orders of magnitude lower than traditional cell-based HTS, representing a trade-off between physiological relevance and sheer scale.

Recent advancements in microfluidic technology have driven the development of innovative platforms, such as the anchored impact platform, which enables high-throughput angiogenesis assessment. For specialized screening, Kim et al. developed the “Anchor-IMPACT” platform for high-throughput antiangiogenic drug screening. While focused on cancer, its application is highly relevant to CKD, where dysregulated angiogenesis is implicated. The platform’s 3D hydrogel-based culture and automated imaging analysis could be directly adapted to screen compounds that normalize pathological angiogenesis in diabetic kidney disease (57). Similarly, paper-based microfluidic analytical devices (μPADs) have emerged as cost-effective and portable tools for high-throughput screening, offering advantages such as ease of fabrication and disposability (58). Furthermore, microfluidic systems have been integrated with advanced analytical techniques like mass spectrometry to enhance the throughput and sensitivity of drug screening assays. For instance, the combination of microfluidic hydrogel droplets with deep learning has been proposed to optimize the crystallization conditions of active pharmaceutical ingredients, demonstrating the potential of microfluidics to streamline drug development processes (59). Additionally, microfluidic platforms have been utilized to screen high lactic acid-producing Bacillus coagulans, showcasing their versatility in various biotechnological applications (60)—relevant to CKD and renal fibrosis through the gut-kidney axis, where microbial metabolites like lactic acid may modulate inflammation and fibrosis, as explored in recent microbiome studies (61). Microfluidic high-throughput drug screening represents a significant advancement in the field of drug discovery, offering numerous advantages over traditional methods. The integration of microfluidic technology with other cutting-edge technologies continues to expand the boundaries of drug screening possibilities, paving the way for more efficient and accurate identification of potential therapeutic agents. Critically, while promising, gaps in multi-organ screening limit full CKD translation—summarizing, 2025 trends emphasize AI-hybrid platforms for enhanced predictive value.

Industry pioneers are bridging these reproducibility and scalability gaps: MIMETAS’ OrganoPlate, a commercially available high-throughput platform (up to 96 wells), supports reproducible kidney models for CKD and fibrosis screening, as seen in their 2024 immunocompetent human kidney-on-a-chip study modeling renal inflammation and immune-mediated injury (32). Companies like Emulate Bio complement this with scalable, modular chips, further driving pharmaceutical adoption.

Table 2 provides an overview of representative organ-on-a-chip models applied to CKD and fibrosis. By bridging *in vitro* and *in vivo* conditions, these platforms not only facilitate targeted drug delivery (e.g., nanoparticle-based systems fabricated under microfluidic control) but also enable regenerative medicine approaches. For instance, organoid-on-chip systems have enhanced nephron differentiation and transporter expression, advancing tissue engineering strategies for CKD. However, reproducibility across laboratories and scalability for pharmaceutical adoption remain unresolved, underscoring the importance of standardization and regulatory guidance.

**TABLE 2:** Organ-on-a-chip models and therapeutic applications in chronic kidney disease (CKD).

OoC model/chip	Cell types/origin	Application	Key finding	Limitations	References
Glomerulus-on-a-chip	iPSC podocytes + human endothelial	Proteinuria modeling	40% nephrin loss in hyperglycemia	No mesangial cells; short-term	(22)
Tubule-on-a-chip	Human PTECs (primary)	Nephrotoxicity testing	Early sensitive responses	Low throughput; viability issues	(39)
Fibrosis model	Tubular + endothelial (human)	Proteinuria-fibrosis	2.5-fold α-SMA via fucosylation	No fibroblasts; model simplification	(24)
Immunocompetent KoC	PTECs + immune cells (human)	Inflammation	Monocyte extravasation upon complement-activated serum	Systemic immunity incomplete	(32)
Multi-organ chip	Liver spheroids (human) + tubules (animal/human)	Drug toxicity	Multi-drug interactions detected	High complexity; cost	(46)

## 5 Interdisciplinary synergies and future directions

### 5.1 Integration with AI and multi-omics

Microfluidic technology has seen widespread application in the biomedical field in recent years, and its integration with AI and multi-omics technologies has further enhanced its potential in diagnostics and therapeutics (Figure 1). With advantages such as high throughput, rapid analysis, low sample volume, and high sensitivity, microfluidic technology is transforming traditional experimental methods. However, challenges remain in the industrialization and commercialization of microfluidic technology, including miniaturization, integration, and intelligentization (62). The application of artificial intelligence in microfluidic technology offers new opportunities for biotechnology research. By combining AI with microfluidic systems, the analysis of large datasets generated from high-throughput and multiplexed microfluidic platforms can be significantly improved (63). This integration not only enhances experimental methods but also reduces costs and scales down operations. Additionally, AI applications in microfluidics include flow cytometry cell classification, cell separation, and more. Despite challenges, the combination of AI methods and microfluidic technology has a significant impact across various fields of biotechnology. In multi-omics research, the integration of AI with microfluidic technology also demonstrates immense potential. Multi-omics technologies can integrate data from genomics, transcriptomics, proteomics, and more, which is crucial for understanding complex health and disease pathways (64). AI, by analyzing large-scale and diverse datasets, can uncover patterns often overlooked by traditional methods, thereby advancing biomarker discovery, understanding drug resistance, and promoting personalized medicine (64). For example, a 2025 Wiley study on microphysiological systems for CKD comorbidity used AI to analyze multi-omics from KoC, predicting fibrosis progression. The combination of microfluidic technology with artificial intelligence and multi-omics not only provides new perspectives in experimental methods and data analysis but also plays a vital role in advancing personalized and precision medicine. This interdisciplinary research offers new hope and possibilities for future medical diagnostics and treatments. Critically, while synergistic, gaps in real-world validation persist—summarizing, 2025 advances position AI-microfluidics as a cornerstone for nephrology precision.

### 5.2 Multi-organ and systemic modeling

Microfluidic technology and multi-organ-on-a-chip (organ-on-a-chip) systems hold significant potential in biomedical research. Microfluidic technology, by manipulating fluids at the microscale, can simulate the *in vivo* microenvironment, offering new avenues for drug screening and disease modeling (55). Multi-organ-on-a-chip technology, by integrating multiple organ models on a single chip, better mimics inter-organ interactions in the body, which is crucial for studying drug metabolism and toxicity (56). In recent years, significant progress has been made in the development of multi-organ-on-a-chip systems using microfluidic technology. For example, researchers have developed a portable and reconfigurable multi-organ platform capable of supporting the functionality and interactions of multiple organs over several weeks (65). Additionally, microfluidic technology has been used to create multi-sensor-integrated organ-on-a-chip platforms with real-time monitoring capabilities, enabling automated and continuous tracking of organ physiological parameters and dynamic responses to drugs (66). In practical applications, microfluidic technology has been employed to construct vascularized and perfused organ-on-a-chip platforms for large-scale drug screening (67). These platforms introduce perfused microvasculature, ensuring the survival of surrounding tissues entirely depends on nutrients delivered through the vessels, thereby better replicating the physiological environment *in vivo*. Furthermore, microfluidic technology has been utilized to develop novel microfluidic models that monitor complex vascular structures and cellular interactions within three-dimensional biological matrices (68). The integration of microfluidic technology and multi-organ-on-a-chip systems provides a powerful tool for biomedical research, enabling more accurate simulation of complex physiological processes in the body and improving the efficiency and precision of drug development.

### 5.3 Ethical and translational challenges

The clinical translation of microfluidic technology involves not only technical challenges but also ethical considerations. As the technology advances, researchers must carefully address ethical issues in study design to ensure scientific rigor and participant safety (69). Ethics committees play a crucial role in this process, ensuring that studies comply with ethical standards and protect participants’ rights (70). Furthermore, the application of microfluidic platforms in drug development has sparked ethical discussions, particularly around efficient small-scale screening that accelerates progress but demands new safeguards for clinical translation—such as addressing biases in patient-derived data, ensuring informed consent for organoid use, and promoting equitable access to these technologies. Researchers must prioritize ethical study design, including transparency in AI algorithms and integrity in reporting, to mitigate risks like data privacy breaches, as per WHO 2024 guidelines. The potential of microfluidic technology in biomedical research is immense, but its clinical translation requires overcoming multiple technical and ethical challenges. By strengthening technical standardization and ethical oversight, microfluidic technology is expected to play an even greater role in future clinical applications.

## 6 Conclusion

Microfluidic technology has significantly advanced our understanding of CKD and renal fibrosis by providing novel platforms for mechanistic studies, diagnostics, and therapeutic development. Through organ-on-a-chip models, high-throughput drug screening, and biomarker detection, microfluidics offers unparalleled precision in disease modeling and intervention. However, challenges remain in standardization, scalability, and regulatory approval for clinical application. Future directions should focus on interdisciplinary collaborations integrating artificial intelligence, multi-omics, and regenerative medicine to optimize microfluidic platforms for CKD research. As microfluidic technology continues to evolve, its potential to bridge bench-to-bedside gaps in nephrology will expand, offering new avenues for early diagnosis, personalized treatment, and improved patient outcomes.
